# Supplementary material for: Low prevalence of bloodstream infection and high blood culture contamination rates in patients with COVID-19
Source: PLoS One. 2020 Nov 23;15(11):e0242533. doi: 10.1371/journal.pone.0242533 (PMC7682817; doi:10.1371/journal.pone.0242533)
Supplement: S1 Table — (DOCX) [file pone.0242533.s001.docx]

**S1 Table**: Distribution of all microorganisms isolated from blood cultures

| **Isolate, episodes including, n** | **COVID-19 group** | **Control group-2020** | **Control group-2019** |
| --- | --- | --- | --- |
| - *Abiotrophia* species | 0 | 2 | 1 |
| - *Acinetobacter* baumannii-group | 0 | 2 | 0 |
| - *Acinetobacter* species, non-baumannii group | 0 | 2 | 0 |
| - *Actinomyces neuii* | 0 | 1 | 0 |
| - *Actinomyces* *odontolyticus* | 0 | 1 | 1 |
| - *Actinomyces* species | 0 | 1 | 2 |
| - *Actinotignum* *schaalii* | 0 | 1 | 3 |
| - *Actinotignum* species | 1 | 3 | 0 |
| - *Aerococcus* sanguinicola | 0 | 0 | 2 |
| - *Aerococcus* urinae | 1 | 3 | 5 |
| - Alpha hemolytic streptococci | 1 | 0 | 2 |
| - *Anaerococcus* species | 0 | 1 | 0 |
| - *Bacteroides fragilis* | 0 | 0 | 1 |
| - *Bacteroides fragilis* group | 2 | 14 | 13 |
| - *Bacteroides* species | 1 | 1 | 1 |
| - *Streptococcus pyogenes* | 2 | 17 | 22 |
| - Beta hemolytic streptococcus group C | 0 | 7 | 8 |
| - Beta hemolytic streptococcus group G | 3 | 12 | 9 |
| - *Bifidobacterium* species | 1 | 0 | 0 |
| - *Campylobacter jejuni* | 0 | 1 | 0 |
| - *Campylobacter ureolyticus* | 0 | 1 | 0 |
| - *Candida albicans* | 2 | 5 | 7 |
| - *Candida glabrata* | 0 | 3 | 6 |
| - *Candida krusei* | 1 | 1 | 0 |
| - *Candida lusitaniae* | 0 | 0 | 1 |
| - *Candida parapsilosis* | 0 | 1 | 0 |
| - *Candida tropicalis* | 0 | 0 | 1 |
| - *Capnocytophaga canimorsus* | 0 | 1 | 1 |
| - *Capnocytophaga* species | 0 | 0 | 1 |
| - *Citrobacter freundii* | 1 | 7 | 4 |
| - *Citrobacter koseri* | 0 | 5 | 4 |
| - *Citrobacter* species | 0 | 2 | 0 |
| - *Clostridium cadaveris* | 0 | 1 | 0 |
| - *Clostridium perfringens* | 1 | 1 | 5 |
| - *Clostridium ramosum* | 0 | 1 | 1 |
| - *Clostridium septicum* | 1 | 0 | 1 |
| - *Clostridium* species | 0 | 2 | 2 |
| - *Eggerthella lenta* | 0 | 3 | 0 |
| - *Eikenella corrodens* | 0 | 0 | 1 |
| - *Enterobacter cloacae* | 4 | 12 | 14 |
| - *Enterobacter cloacae* ESBL | 1 | 0 | 0 |
| - *Enterococcus avium* | 1 | 1 | 1 |
| - *Enterococcus casseliflavus* | 0 | 3 | 0 |
| - *Enterococcus faecalis* | 17 | 36 | 40 |
| - *Enterococcus faecium* | 5 | 6 | 31 |
| - *Enterococcus faecium* VRE | 0 | 0 | 1 |
| - *Enterococcus gallinarum* | 0 | 0 | 1 |
| - *Enterococcus* species | 0 | 0 | 1 |
| - *Escherichia coli* | 28 | 197 | 210 |
| - *Escherichia coli* ESBL | 6 | 16 | 32 |
| - *Fusobacterium necrophorum* | 0 | 0 | 3 |
| - *Fusobacterium nucleatum* | 0 | 0 | 2 |
| - *Gardnerella vaginalis* | 0 | 1 | 1 |
| - *Gemella* species | 0 | 4 | 0 |
| - *Globicatella sanguinis* | 0 | 0 | 1 |
| - Gram negative coccus, unspecified | 0 | 1 | 1 |
| - Gram negative rod, unspecified | 0 | 0 | 7 |
| - Gram negative rod, *Enterobacterales* | 0 | 0 | 1 |
| - Gram positive coccus, anaerobe, unspecified | 0 | 0 | 1 |
| - Gram positive coccus, suspected *enterococcus* or *streptococcus* | 0 | 0 | 2 |
| - Gram positive coccus, suspected *pneumococcus* | 0 | 1 | 0 |
| - Gram positive rod, anaerobe | 1 | 1 | 2 |
| - *Granulicatella adiacens* | 1 | 2 | 3 |
| - Group B *streptococcus* (*Streptococcus agalactiae*) | 1 | 11 | 11 |
| - *Haemophilus influenzae* | 0 | 1 | 6 |
| - *Hafnia alvei* | 0 | 1 | 0 |
| - Yeast, unspecified | 0 | 1 | 0 |
| - *Klebsiella (Enterobacter) aerogenes* | 2 | 2 | 2 |
| - *Klebsiella oxytoca* | 2 | 11 | 16 |
| - *Klebsiella pneumoniae* | 8 | 27 | 26 |
| - *Klebsiella pneumoniae* ESBL | 0 | 4 | 8 |
| - *Klebsiella variicola* | 1 | 6 | 8 |
| - *Lactococcus* species | 0 | 1 | 1 |
| - *Leptotrichia* species | 0 | 0 | 1 |
| - *Listeria monocytogenes* | 0 | 3 | 0 |
| - *Moraxella catarrhalis* | 0 | 1 | 1 |
| - *Moraxella* species | 0 | 1 | 2 |
| - *Morganella morganii* | 0 | 4 | 0 |
| - *Neisseria meningitidis* | 0 | 1 | 1 |
| - *Neisseria* species | 0 | 0 | 2 |
| - *Pantoea* species | 0 | 2 | 0 |
| - *Parvimonas micra* | 0 | 3 | 5 |
| - *Peptoniphilus* species | 0 | 4 | 1 |
| - *Prevotella* species | 0 | 1 | 1 |
| - *Proteus mirabilis* | 1 | 13 | 13 |
| - *Proteus vulgaris* | 0 | 0 | 1 |
| - *Proteus vulgaris* group | 1 | 1 | 2 |
| - *Providencia rettgeri* | 1 | 0 | 2 |
| - *Providencia* species | 0 | 0 | 1 |
| - *Pseudomonas aeruginosa* | 7 | 13 | 14 |
| - *Ruminococcus* species | 0 | 1 | 0 |
| - *Salmonella dublin* | 1 | 0 | 0 |
| - *Salmonella enterica* | 0 | 1 | 0 |
| - *Salmonella enteritidis* | 0 | 1 | 0 |
| - *Salmonella java* | 0 | 0 | 1 |
| - *Salmonella paratyphi* A | 0 | 2 | 1 |
| - *Salmonella* species | 0 | 2 | 5 |
| - *Salmonella stanley* | 0 | 0 | 1 |
| - *Serratia marcescens* | 1 | 7 | 6 |
| - *Serratia* species | 0 | 0 | 1 |
| - *Staphylococcus aureus* | 41 | 121 | 158 |
| - *Staphylococcus aureus* MRSA | 3 | 3 | 4 |
| - *Stenotrophomonas maltophilia* | 1 | 3 | 1 |
| - *Streptococcus anginosus* *(milleri)* group | 5 | 19 | 16 |
| - *Streptococcus bovis* group | 2 | 3 | 6 |
| - *Streptococcus mitis* group | 6 | 21 | 16 |
| - *Streptococcus mutans* group | 1 | 1 | 0 |
| - *Streptococcus pneumoniae* | 3 | 21 | 51 |
| - *Streptococcus salivarius* group | 2 | 8 | 7 |
| - *Streptococcus sanguinis* group | 5 | 12 | 16 |
| - *Veillonella* species | 0 | 2 | 0 |
| - *Yersinia enterocolitica* non-O3/O9 | 0 | 1 | 0 |
| - *Corynebacterium* species | 0 | 0 | 1 |
| - *Cutibacterium (Propionibacterium) acnes* | 0 | 0 | 1 |
| - Gram positive rod, unspecified | 0 | 0 | 3 |
| - Coagulase negative staphylococcus | 27 | 16 | 31 |
| - *Staphylococcus epidermidis* | 21 | 38 | 25 |
| - *Staphylococcus lugdunensis* | 1 | 1 | 2 |
| - *Bacillus cereus* | 0 | 0 | 1 |
| - *Lactobacillus* species | 0 | 1 | 0 |
| - *Corynebacterium striatum* | 0 | 0 | 1 |
| **Total** | 226 | 781 | 940 |
