# Supplementary material for: Low prevalence of bloodstream infection and high blood culture contamination rates in patients with COVID-19
Source: PLoS One. 2020 Nov 23;15(11):e0242533. doi: 10.1371/journal.pone.0242533 (PMC7682817; doi:10.1371/journal.pone.0242533)
Supplement: Attachment — Submitted filename: LAST Reply to the Editor and Reviewers 200917 .docx [file pone.0242533.s002.docx]

**Reply to the Editor and Reviewers’ Comments**

**Additional Editor Comments:**

1. In the absence of clinical data, the length of the manuscript should be shortened and the lack of clinical data should be acknowledged as an important limitation.

**As the Editor suggested the original version of the manuscript is shortened and the limitation with the lack of clinical data is included in the discussion section as follows: “****The present study has important limitations. First, we did not have access to base-line clinical data such as co-morbidity, disease duration and treatment of patient in all three groups. Therefore, assessment of the impact of differences in patient characteristics on the blood culture results could not be analyzed….”**

2. Include numbers and proportions of patients admitted during the study time-frame (in 2020) that underwent SARS-CoV-2 testing. Also state whether testing for SARS-CoV-2 was done on all admitted patients or only those who presented with symptoms, to help understand if the control group represented SARS-CoV2 negative patients, or a mix of tested-negative and patients not tested. If possible, briefly describe the COVID-19 care model and infection control practice in the study period. This would help better understand the context for higher contamination rates.

**The contemporary group in 2020 was heterogenous in regard to SARS-COV-2 testing. The number of patients admitted to the hospital during the study periods is not known to us, and therefore the proportion of tested patients is unknown. The testing routine for SARS-COV-2 varied during the study period. In the beginning of the pandemic, testing was only done in patients with symptoms consistent with COVID-19 and not all admitted patients. In the present study, the control group therefore consisted of a mix of negative patients and patients not tested. However, all patients with COVID-19-like symtoms were tested. Therefore, it is reasonable to assume that the patients not tested for SARS-COV-2 did not have clinical findings of COVID-19.**

**The question on the COVID-19 care model and infection control practice in the study period is very relevant. However, the care of the COVID-19 patients was not consistent during the early phase of the pandemic in Sweden as in other countries and it is difficult to describe shortly in the current manuscript. We believe it is also out of the scope of the current study.**

3. Please include the blood culturing frequency (relative to the number of patients or patient-days) for the three cohorts. The number of contaminated blood cultures may also be related to more blood cultures being obtained.

**The study included patient samples from six tertiary care hospitals. Therefore, it is rather difficult to obtain exact numbers of patients treated during this period.**

**The COVID-19 patients were in total 2240 (27%), of all patients (8262) sampled for blood cultures during the study period. The total number of blood cultured patient during the study period, 8262, is an increase of 1421 (20%), compared to the same period last year (total 6841 patients). Normally we have a yearly 10% increase in numbers of our blood culture bottles at Karolinska without any change in contamination rates. Therefore it is highly unlikely that the increase in number of contaminated blood cultures is related to more blood cultures being obtained.**

4. Related to the point above, please be careful with the use of terms “positivity” and “bacteremia rate” both when describing your own results and when comparing with the published literature (e.g., New York data). I believe that in this context, positivity is the positive blood cultures/total number of episodes whereas bacteremia rates are positive blood cultures/total patients. These terms should be described and used consistently.

**In the present study, a previous established algorithm was used to determine isolates as clinically relevant or contamination. Therefore, we used “positivity” to denote overall positive blood culture episodes, where as “clinically relevant” is used to describe isolates which represent true bacteremia. As the analyses in our study is based on episodes, we avoid the term bacteremia and instead use “positive episodes” and “episodes with clinically relevant growth” in the revised version.**

5. Both reviewers have commented on the lack of relevant clinical data. While including clinical data might be out of the scope of your study, are you able to include data on two important variables: 1) hospital location, and 2) time from admission as relevant clinical variables that would provide important information. Specifically, blood cultures obtained in the ED setting are well described to have high rates of contamination so location where blood cultures were obtained particularly ED vs inpatient should be reported. Second, as pointed out be reviewer 2, it would be clinically useful to compare positivity for contaminants and non-contaminants by time since admission – you could report this stratified by within one week from admission, and after one week from admission.

**We acknowledge the lack of clinical data. As the reviewers suggested, the data on blood culture results in relation to hospital locations is included and the data on the following three groups are presented in the revised version: Emergency department, intensive care unit, and others. The data is presented in a new table (Table 3) as follows:**

**Table 3.** Positive episodes and proportion of contamination in blood cultures from different hospital locations.

| **Hospital location** | **COVID-19** | | **Control group 2020** | | **Control group 2019** | |
| --- | --- | --- | --- | --- | --- | --- |
|  | **Positive episodes** | **Contaminant (% of positive)** | **Positive episodes** | **Contaminant (% of positive)** | **Positive episodes** | **Contaminant (% of positive)** |
| **Emergency department** | 183 | 112 (61.2) | 415 | 130 (31.3) | 352 | 110 (31.2) |
| **Intensive care unit** | 108 | 70 (64.8) | 32 | 18 (56.2) | 30 | 16 (53.3) |
| **Other hospital locations** | 142 | 73 (58.9) | 568 | 182 (32.0) | 771 | 228 (29.6) |

**The information on time since admission is unfortunately not available, as we did not have access to the patient records including time that the patient was admitted to the hospital. We had only the time of BC sampling as it is available in our laboratory information system.**

6. Was any statistical testing done for time to positivity, and proportion positive by time period?

**No statistical testing was done for time to positivity and proportion positive by time period.**

Journal requirements:

When submitting your revision, we need you to address these additional requirements.

1. Please ensure that your manuscript meets PLOS ONE's style requirements, including those for file naming. The PLOS ONE style templates can be found at

[https://journals.plos.org/plosone/s/file?id=wjVg/PLOSOne_formatting_sample_main_body.pdf](https://eur01.safelinks.protection.outlook.com/?url=https%3A%2F%2Fjournals.plos.org%2Fplosone%2Fs%2Ffile%3Fid%3DwjVg%2FPLOSOne_formatting_sample_main_body.pdf&data=02%7C01%7Cdavid.yu%40ki.se%7Cf1876cab304646661cf908d84784131d%7Cbff7eef1cf4b4f32be3da1dda043c05d%7C0%7C0%7C637337980484195022&sdata=I5VzRnEVUGNYtdslIGYcicUxjMwn1vCxj%2FdMxoFzens%3D&reserved=0) and

[https://journals.plos.org/plosone/s/file?id=ba62/PLOSOne_formatting_sample_title_authors_affiliations.pdf](https://eur01.safelinks.protection.outlook.com/?url=https%3A%2F%2Fjournals.plos.org%2Fplosone%2Fs%2Ffile%3Fid%3Dba62%2FPLOSOne_formatting_sample_title_authors_affiliations.pdf&data=02%7C01%7Cdavid.yu%40ki.se%7Cf1876cab304646661cf908d84784131d%7Cbff7eef1cf4b4f32be3da1dda043c05d%7C0%7C0%7C637337980484195022&sdata=KM3D%2F8uEPbOCbUc%2FiPEEUm53wMm6RLgyLWfi1UQCgPQ%3D&reserved=0)

**We followed the PLOS ONE's style requirements.**

2. Please include the date(s) on which you accessed the laboratory information system to obtain the data used in your study.

**All data was retrieved from the LIS on 30^th^ of April 2020.**

3. PLOS ONE requires experimental methods to be described in enough detail to allow suitably skilled investigators to fully replicate and evaluate your study.

**The study is based on data collection from the LIS. The study can easily be reproduced by a similar search in any LIS including ours.**

See [https://journals.plos.org/plosone/s/submission-guidelines#loc-materials-and-methods](https://eur01.safelinks.protection.outlook.com/?url=https%3A%2F%2Fjournals.plos.org%2Fplosone%2Fs%2Fsubmission-guidelines%23loc-materials-and-methods&data=02%7C01%7Cdavid.yu%40ki.se%7Cf1876cab304646661cf908d84784131d%7Cbff7eef1cf4b4f32be3da1dda043c05d%7C0%7C0%7C637337980484205017&sdata=cFIUiC%2FRAjb8qVbOhMVTL%2F%2FXJI8LCxAM5vJ1vEanTXY%3D&reserved=0) for more information. To comply with PLOS ONE submission guidelines, in your Methods section, please provide a more detailed description of microorganism identification and antimicrobial susceptibility methodology.

4. We note that you have included the phrase “data not shown” in your manuscript. Unfortunately, this does not meet our data sharing requirements. PLOS does not permit references to inaccessible data. We require that authors provide all relevant data within the paper, Supporting Information files, or in an acceptable, public repository. Please add a citation to support this phrase or upload the data that corresponds with these findings to a stable repository (such as Figshare or Dryad) and provide and URLs, DOIs, or accession numbers that may be used to access these data. Or, if the data are not a core part of the research being presented in your study, we ask that you remove the phrase that refers to these data.

**As the data that were not shown was not a part of the core results, we have decided to remove it from the revised version of the manuscript.**

5. Please include your tables as part of your main manuscript and remove the individual files.

Please note that supplementary tables should be uploaded as separate "supporting information" files.

**Thank you, we will submit the tables and supplementary tables as suggested.**

**Reply to Reviewers’ comments**

**Comments to the Author**

1. Is the manuscript technically sound, and do the data support the conclusions?

The manuscript must describe a technically sound piece of scientific research with data that supports the conclusions. Experiments must have been conducted rigorously, with appropriate controls, replication, and sample sizes. The conclusions must be drawn appropriately based on the data presented.

Reviewer #1: Yes

Reviewer #2: Yes

2. Has the statistical analysis been performed appropriately and rigorously?

Reviewer #1: Yes

Reviewer #2: Yes

3. Have the authors made all data underlying the findings in their manuscript fully available?

The [PLOS Data policy](https://eur01.safelinks.protection.outlook.com/?url=http%3A%2F%2Fwww.plosone.org%2Fstatic%2Fpolicies.action%23sharing&data=02%7C01%7Cdavid.yu%40ki.se%7Cf1876cab304646661cf908d84784131d%7Cbff7eef1cf4b4f32be3da1dda043c05d%7C0%7C0%7C637337980484205017&sdata=XXc%2FtpvhHhsZKfrlUxMlkyKuQretqbqD%2B9AZESciKTU%3D&reserved=0) requires authors to make all data underlying the findings described in their manuscript fully available without restriction, with rare exception (please refer to the Data Availability Statement in the manuscript PDF file). The data should be provided as part of the manuscript or its supporting information, or deposited to a public repository. For example, in addition to summary statistics, the data points behind means, medians and variance measures should be available. If there are restrictions on publicly sharing data—e.g. participant privacy or use of data from a third party—those must be specified.

Reviewer #1: Yes

Reviewer #2: Yes

4. Is the manuscript presented in an intelligible fashion and written in standard English?

PLOS ONE does not copyedit accepted manuscripts, so the language in submitted articles must be clear, correct, and unambiguous. Any typographical or grammatical errors should be corrected at revision, so please note any specific errors here.

Reviewer #1: Yes

Reviewer #2: Yes

5. Review Comments to the Author

Please use the space provided to explain your answers to the questions above. You may also include additional comments for the author, including concerns about dual publication, research ethics, or publication ethics. (Please upload your review as an attachment if it exceeds 20,000 characters)

Reviewer #1: a bit lengthy and wordy for the data being presented, and could be presented much more concisely while writing is grammatically accurate, the English is a little clunky clinical data would be useful given some of the demographic discrepancies (greater M > F ratio in COVID group) would favor medians instead of means would be consistent with abbreviations, especially with bacteria names

**As the Reviewer suggested the revised version of the manuscript is shortened and medians were used instead of means.**

line 181 - Enterobacterales isn't a species

**Corrected**

might be helpful to note numbers of repeat episodes

**Done**

any difference in pathogens isolated by hospital day? ie were contaminants more likely to be isolated on admission vs later in the hospitalization which may have represented a clinically relevant pathogen?

**Unfortunately, we did not have access to patient journals. The revised manuscript includes data on hospital location when BC sampling was performed. As it is shown in Table 3, both control groups had higher contamination rates in ICU than in emergency departments and other clinics. However, the contamination rates were similar among emergency departments, ICU and other clinics for patients with COVID-19 which indicates that the high rate of contamination in patients with COVID-19 is probably related to difficulties in optimal blood culture sampling with the protective equipment and stress level.**

Reviewer #2: Thank you for the opportunity to review this interesting and well written manuscript on secondary bacterial BSI in COVD-19 patients. This study adds important information to the literature by providing comparison to non-COVID patients both during the pandemic and historically. In my opinion, this is an important comparison that has not yet been made properly and begins to address some of the missing gaps in knowledge surrounding COVID-19 and bacterial / fungal infections.

**We thank the Reviewer for acknowledging the importance of our study.**

One area that could be stronger in the discussion is the nature of secondary infection in COVID-19 and an exploration of whether it is directly attributable to COVID-19 or a consequence of increased healthcare exposure and pressures on services.

**The question of the underlying cause for lower bacteremia rates and higher contamination rates in COVID-19 is highly relevant. However, the results of the present study are not sufficient to determine the underlying cause as clinical parameters were lacking.**

Abstract
No comments

Introduction
1. Secondary bacterial infections in influenza often refers to respiratory tract infection. The authors of this study have focused on blood culture results. This makes comparison between influenza and COVID-19 in this case challenging.

**We agree with the Reviewer that bacterial LRTI are the major secondary bacterial infections after viral RTI. However, the aim of the current study was to analyze the association between COVID-19 and BSI.**

Method:
1. Were all patients admitted during March and April tested for SARS-CoV-2? If patients with high clinical suspicion were not routinely tested for example this may have led to overlap in the SARS-CoV-2 and negative control group. Similarly, false negatives / positive results may have occurred. This should be acknowledged as a limitation of the study.

**This relevant question was also raised by the Editor. In the present study, during the 2020 study period, the testing routine for SARS-COV-2 was not consistent. During the pandemic, SARS-COV-2 testing was initially performed only for patients with clinical signs of COVID-19. Therefore, there is a possibility that the negative control group might have included patients that had been positive if a ”test all” strategy had been employed. However, as virtually all patients with clinical signs were tested, it is possible to assume that the control group-2020 represent patients without clinical significant COVID-19.**

2. How were contaminant organisms determined?

**There is no gold standard definition of contaminants in blood cultures. We used a modified version of a previously published algorithm by our group as well as others (Yu et al 2020; Bekeris et al., 2005; Dawson, 2014).**

3. Were control groups matched in any way? If not how were the control samples selected?

**Control groups were not matched. Control samples 2020 were taken during the same study period as SARS-COV-2 samples.**

Results:
1. What was the total number of COVID-19 positive patients from which the blood culture pool were selected during the study time period?

**The total numbers of COVID-19 patients under the study period were 7617.**

2. Doe the authors have data to describe the setting where blood cultures were taken for these patients? For example, were line associated cultures more common in COVID patients? Were more cultures performed in critical care?

**Whether sampling from peripheral or central vein catheters were not documented consistently. However, we described the numbers of positive samples from the three hospital locations namely emergency department, intensive care unit, and others in the revised version of the manuscript.**

3. Can the authors provide any information of antimicrobial prescribing trends during the time periods described?

**This is an important question. The study included six tertiary care hospitals and the data on antimicrobial prescribing trends during the time periods was not available since we did not have access to patient journals.**

Discussion:
1. An important point is the reason for contamination of blood cultures. This should be explored further. Is this the nature of PPE use, reduced hand hygiene, or due to staffing issues during expanded critical care capacity?

**The underlying reasons for higher contamination rates are probably multifactorial and consist of all of the above-mentioned problems. Detailed analysis of these factors could not be made, and they are out of the scope of the study.**

2. Furthermore, could the shift in observed species be due to reductions in elective intra-abdominal and urinary operations / procedures being performed during this period?

**The patient profile in these six hospitals and in almost all hospitals in Sweden and in other countries has indeed changed dramatically as the Reviewer indicated. It is possible that the reduction of elective patients reflects the microorganisms isolated from BC. However, this factor was not analyzed in the current study.**

3. It would also be interesting to known whether the absolute number of patients with COVID in the healthcare system during this time. For example, could it be that Gram-negative infections were missed as fever was put down to COVID and less cultures were actually performed?

**The COVID-19 patients were in total 2240/8262 (27%), of all patients sampled for blood cultures during the study period. The total number of blood cultured patients during the study period were 8262 and 20% higher compared to the same period last year (total 6841 patients in 2019).**

**The total number of COVID-19 positive patients during the study period was 7617, which includes also non-hospitalized patients with mild symptoms. It is reasonable to assume that the results are influenced by the shift in patient profiles, as mentioned above. However, our data precludes conclusions regarding potential effects from clinical sampling strategies.**

4. Another important limitation of this study that should be acknowledged and discussed is that the authors have not looked at other microbiological cultures. For example, a major concern during COVID-19 has been the reporting of bacterial/fungal respiratory infection. The authors are unable to comment on secondary infections such as HAP and VAP given their focus on blood cultures only.

**We thank the reviewer for acknowledging this limitation, which have been added to the discussion section in the revised manuscript: “Third, as the present study focused on BSI in COVID-19, data regarding other cultures were not analyzed, which precluded the analysis of other secondary infections such as pneumonia. “**

5. Furthermore, by not providing clinical details, it is not possible to know whether patients with and without COVID-19 are matched or different cohorts (e.g. number of critically unwell patients, number of line days per patient, number of patients with respiratory versus GI pathology etc.).

**The lack of clinical data has been emphasized as an important limitation in the revised manuscript.**

6. One area that could come through stronger in the discussion is the nature of secondary infection in COVID-19 and exploration of whether it is directly attributable to COVID-19 or a consequence of increased healthcare exposure and pressures on services. For example, are these infections similar to routine HCAI’s associated with ICU / increased line days? And are we seeing more of them due to expanded ICU capacity during this period?

**This is an important point, however the cause of secondary infection in COVID-19 is difficult to elucidate from the parameters included in the present study. Most likely, it is multifactorial and requires an analysis on both a microbiological, clinical, and logistical level which is out of the scope of this study.**

6. PLOS authors have the option to publish the peer review history of their article ([what does this mean?](https://eur01.safelinks.protection.outlook.com/?url=https%3A%2F%2Fjournals.plos.org%2Fplosone%2Fs%2Feditorial-and-peer-review-process%23loc-peer-review-history&data=02%7C01%7Cdavid.yu%40ki.se%7Cf1876cab304646661cf908d84784131d%7Cbff7eef1cf4b4f32be3da1dda043c05d%7C0%7C0%7C637337980484215009&sdata=2ghyqlp6uBNL29pq7Utk301RDBIL%2F5NSzjarSlaJ51E%3D&reserved=0)). If published, this will include your full peer review and any attached files.


If you choose “no”, your identity will remain anonymous but your review may still be made public.


**Do you want your identity to be public for this peer review?** For information about this choice, including consent withdrawal, please see our [Privacy Policy](https://eur01.safelinks.protection.outlook.com/?url=https%3A%2F%2Fwww.plos.org%2Fprivacy-policy&data=02%7C01%7Cdavid.yu%40ki.se%7Cf1876cab304646661cf908d84784131d%7Cbff7eef1cf4b4f32be3da1dda043c05d%7C0%7C0%7C637337980484215009&sdata=os7CVDnT4IgKoC3z2PrucyAaly7G2pfU0aGxPiuszZs%3D&reserved=0).

Reviewer #1: No

Reviewer #2: No

[NOTE: If reviewer comments were submitted as an attachment file, they will be attached to this email and accessible via the submission site. Please log into your account, locate the manuscript record, and check for the action link "View Attachments". If this link does not appear, there are no attachment files.]

While revising your submission, please upload your figure files to the Preflight Analysis and Conversion Engine (PACE) digital diagnostic tool, [https://pacev2.apexcovantage.com/](https://eur01.safelinks.protection.outlook.com/?url=https%3A%2F%2Fpacev2.apexcovantage.com%2F&data=02%7C01%7Cdavid.yu%40ki.se%7Cf1876cab304646661cf908d84784131d%7Cbff7eef1cf4b4f32be3da1dda043c05d%7C0%7C0%7C637337980484225005&sdata=dMBPQ47VhFba9UWb6%2BCoGEH6gaRC88DUlilwNmS6k3E%3D&reserved=0). PACE helps ensure that figures meet PLOS requirements. To use PACE, you must first register as a user. Registration is free. Then, login and navigate to the UPLOAD tab, where you will find detailed instructions on how to use the tool. If you encounter any issues or have any questions when using PACE, please email PLOS at [figures@plos.org](mailto:figures@plos.org). Please note that Supporting Information files do not need this step.

*In compliance with data protection regulations, you may request that we remove your personal registration details at any time.* [*(Remove my information/details)*](https://eur01.safelinks.protection.outlook.com/?url=https%3A%2F%2Fwww.editorialmanager.com%2Fpone%2Flogin.asp%3Fa%3Dr&data=02%7C01%7Cdavid.yu%40ki.se%7Cf1876cab304646661cf908d84784131d%7Cbff7eef1cf4b4f32be3da1dda043c05d%7C0%7C0%7C637337980484225005&sdata=hNXLecoTSRfk4BqsFYZau5Lcgw0d10in8Sk0uKYFMyM%3D&reserved=0)*. Please contact the publication office if you have any questions.*

*När du skickar e-post till Karolinska Institutet (KI) innebär detta att KI kommer att behandla dina personuppgifter.* [Här finns information om hur KI behandlar personuppgifter](https://eur01.safelinks.protection.outlook.com/?url=https%3A%2F%2Fki.se%2Fmedarbetare%2Fintegritetsskyddspolicy&data=02%7C01%7Cdavid.yu%40ki.se%7Cf1876cab304646661cf908d84784131d%7Cbff7eef1cf4b4f32be3da1dda043c05d%7C0%7C0%7C637337980484234999&sdata=%2Foe87SZywM6IffokUtjVhuzsklIqyjbiiriVrMY4Psw%3D&reserved=0).

*Sending email to Karolinska Institutet (KI) will result in KI processing your personal data.* [You can read more about KI’s processing of personal data here](https://ki.se/en/staff/data-protection-policy).
